# Is One Year Enough? Extended Adjuvant Dabrafenib Plus Trametinib for Chinese Patients With Resected Stage III Melanoma

**DOI:** 10.1111/1346-8138.17779

**Published:** 2025-05-14

**Authors:** Dong‐Dong Jia, Yu Xu, Zhi‐Wu Ren, Lin‐Qing Li, Lei Zhang, Yang Li, Li‐Shu Lou, Wei‐Tao Yao, Zhe Liu, Xian‐An Li, Ji‐Long Yang, Yong Chen, Tao Li

**Affiliations:** ^1^ Department of Bone and Soft‐Tissue Surgery Zhejiang Cancer Hospital, Hangzhou Institute of Medicine (HIM), Chinese Academy of Sciences Zhejiang Hangzhou China; ^2^ Department of Musculoskeletal Surgery Fudan University Shanghai Cancer Center, Shanghai Medical College, Fudan University Shanghai China; ^3^ Department of Bone and Soft Tissue Tumor Tianjin Medical University Cancer Institute and Hospital Tianjin China; ^4^ Department of Bone and Soft‐Tissue Surgery Hunan Cancer Hospital Changsha Hunan China; ^5^ Bone and Soft Tissue Oncology Department Jiangxi Cancer Hospital Nanchang Jiangxi China; ^6^ Department of Bone and Soft Tissue Henan Cancer Hospital Zhengzhou Henan China; ^7^ Zhejiang Cancer Hospital Hangzhou Institute of Medicine (HIM), Chinese Academy of Sciences Zhejiang Hangzhou China

**Keywords:** chinese patients, dabrafenib plus trametinib, extended adjuvant therapy, relapse‐free survival, stage III melanoma

## Abstract

**Background:**

Pathogenic BRAF mutations drive constitutive MAPK pathway activation in melanoma, and targeted therapies with dabrafenib plus trametinib have improved outcomes in the adjuvant setting. However, the optimal duration of adjuvant therapy remains unclear. This retrospective study examined whether extending dabrafenib plus trametinib beyond 1 year offers additional clinical benefit in Chinese patients with resected Stage III melanoma.

**Methods:**

Medical records from six centers were reviewed for adults with BRAF V600E/K–positive, completely resected Stage III melanoma who received at least 12 months of adjuvant dabrafenib plus trametinib. Patients were divided into a 1‐year therapy group and a more‐than‐1‐year therapy group. Relapse‐free survival (RFS) was the primary end point; adverse events were also assessed.

**Results:**

Of the 122 patients included, 77 received more than 1 year of adjuvant therapy. The more‐than‐1‐year group experienced significantly better RFS (log‐rank *p* = 0.04), and longer therapy independently reduced recurrence risk in multivariate analysis (HR, 2.42; *p* = 0.035). Adverse event profiles did not differ between groups, and toxicity‐related treatment modifications occurred primarily within the first year.

**Conclusions:**

Extending dabrafenib plus trametinib beyond 1 year may provide improved RFS without increasing toxicity. Further prospective trials are warranted to confirm the impact on overall survival and identify optimal patient subsets for prolonged therapy.

## 1 Introduction

Pathogenic BRAF mutations, present in approximately 61% of cutaneous melanomas and 14% of acral melanomas, drive the constitutive activation of the MAP kinase signaling pathway [1, 2]. The identification of BRAF V600E and V600K as predominant variants facilitated the development of targeted therapies with small‐molecule inhibitors [3]. Improved survival with BRAF/MEK inhibitors in unresectable metastatic melanoma has supported their evaluation in the adjuvant setting [4].

The pivotal COMBI‐AD trial demonstrated the efficacy of 12 months of adjuvant dabrafenib plus trametinib (BRAF/MEK inhibitors) compared to placebo in patients with resected Stage III melanoma harboring BRAF V600E/K mutations [5]. After 10 years of follow‐up, dabrafenib plus trametinib improved relapse‐free survival (RFS) (hazard ratio [HR], 0.52) and distant metastasis‐free survival (HR, 0.56) in Stage III melanoma, with a 20% nonsignificant overall survival (OS) benefit [6]. In the adjuvant targeted therapy (TT) group, RFS rates were 88% at 1 year, 67% at 2 years, and 58% at 3 years [5]. Only a few RFS events occurred beyond 3 years, suggesting a potential plateau [7]. The superior prevention of early recurrences with TT compared to PD‐1 inhibitors can be inferred from the results of the COMBI‐AD and KEYNOTE‐054 trials [5, 8]. Frequent recurrence of melanoma within the first year after discontinuing adjuvant TT was observed. This phenomenon was also noted in our study, which focused on Chinese patients with Stage III melanoma receiving targeted adjuvant therapy [9]. These observations suggest that a 12‐month treatment duration may be insufficient. Many physicians extend treatment beyond 1 year despite lacking evidence to support additional benefits.

In this retrospective study, we compared the efficacy of 1 year versus extended adjuvant therapy with dabrafenib combined with trametinib in Chinese patients from six centers diagnosed with Stage III malignant melanoma harboring BRAF mutations.

## 2 Methods

### 2.1 Patients

A retrospective cohort of melanoma patients who received adjuvant TT at Zhejiang Cancer Hospital, Fudan University Shanghai Cancer Center, Tianjin Cancer Hospital, Hunan Cancer Hospital, Jiangxi Cancer Hospital, or Henan Cancer Hospital between August 2019 and September 2024 was identified. All patient data were obtained from the hospitals' databases and subsequently archived in an internal computerized database.

Inclusion criteria were as follows: age ≥ 18 years, a histologically confirmed diagnosis of cutaneous or acral melanoma harboring a BRAFV600E or BRAFV600K mutation, complete resection of the primary tumor and any metastatic lesions (including regional lymph‐node dissection, if applicable) performed within 13 weeks prior to initiation of adjuvant therapy, and receipt of combined trametinib and dabrafenib (adjuvant TT) for at least 12 months. Exclusion criteria included any prior systemic therapy or radiotherapy for melanoma, clinical or radiological evidence of residual disease following surgery, recurrence or discontinuation of adjuvant TT within 12 months, or a diagnosis of a malignancy other than melanoma.

### 2.2 Primary and Secondary End Points

The primary end point of this study was RFS, defined as the interval from the first dose of adjuvant dabrafenib + trametinib to whichever of the following occurred first: (i) radiologically or pathologically confirmed local, regional, or distant recurrence; (ii) appearance of a new lesion (see definition below); or (iii) death from any cause. A new lesion was defined as follows: (a) a previously unseen non‐nodal lesion ≥ 10 mm in longest diameter; (b) a lymph‐node lesion with short axis ≥ 15 mm; or (c) an unequivocally positive lesion detected on CT, MRI, or PET‐CT. Lesions smaller than these size thresholds were also counted if biopsy‐proven malignant.

Radiologic assessments followed RECIST v1.1. Baseline staging included contrast‐enhanced CT of the chest and, when clinically indicated, CT or MRI of other relevant regions, plus a brain MRI performed within 4 weeks before the start of adjuvant therapy. Follow‐up imaging was conducted roughly every 3 months during the first 2 years and every 6 months thereafter.

Safety assessments included the collection of adverse events, clinical laboratory test results, physical examination findings, and vital signs. The severity of each adverse event and its relationship to the investigational drug were evaluated according to the Common Terminology Criteria for Adverse Events (CTCAE).

### 2.3 Statistical Analysis

All eligible patients were included in the analysis. Clinicopathological factors were summarized using descriptive statistics, and group comparisons were made using the Chi‐square test. RFS was estimated by the Kaplan–Meier method. HRs and their 95% confidence intervals (CIs) were calculated using a Cox proportional hazards model, and survival distributions were compared using log‐rank tests.

Associations between predictor variables and time‐to‐event outcomes were investigated through univariate and multivariate Cox proportional hazards analyses using SPSS (Version 25). Statistical significance was defined as a *p*‐value < 0.05.

## 3 Results

### 3.1 Baseline Characteristics

Table 1 presents the baseline characteristics of the study population. All patients were Chinese, and 49 (40.2%) were male. The median age was 54 years (range, 26–84 years). A total of 84 (68.9%) patients had cutaneous melanoma and 34 (27.9%) had acral melanoma. Except for two patients with a BRAF V600K mutation, all other patients had a BRAF V600E mutation. The majority of patients had Stage IIIB (44 [36.1%]) or Stage IIIC (55 [45.1%]). Patients were recruited from the following centers: 45 (36.9%) from Zhejiang Cancer Hospital, 56 (45.9%) from Fudan University Shanghai Cancer Center, 4 (3.3%) from Tianjin Cancer Hospital, 12 (9.8%) from Hunan Cancer Hospital, 2 (1.6%) from Jiangxi Cancer Hospital, and 3 (2.5%) from Henan Cancer Hospital.

**TABLE 1:** Baseline demographics and disease characteristics.

	1‐year adjuvant therapy group (*n* = 45)	More‐than‐1‐year adjuvant therapy group (*n* = 77)	*p*
Sex			0.977
Male	18 (40%)	31 (40.26%)	
Female	27 (60%)	46 (59.74%)	
Age			0.735
< 60 year	30 (66.67%)	49 (63.64%)	
≥ 60 year	15 (33.33%)	28 (36.36%)	
Disease stage			0.503
III B	19 (42.22%)	25 (32.47%)	
III C	20 (44.44%)	35 (45.45%)	
III D	3 (6.67%)	11 (14.29%)	
III unknown	3 (6.67%)	6 (7.79%)	
Subtype			0.841
Acral	12 (26.67%)	52 (67.53%)	
Cutaneous	32 (71.11%)	22 (28.57%)	
Unknown	1 (2.22%)	3 (3.90%)	
Center			0.117
Zhejiang	15 (33.33%)	30 (38.96%)	
Shanghai	20 (44.44%)	36 (46.75%)	
Hunan	4 (8.89%)	8 (10.39%)	
Tianjin	3 (6.67%)	1 (1.30%)	
Henan	3 (6.67%)	0 (0%)	
Jiangxi	0 (0%)	2 (2.60%)	

The 1‐year adjuvant therapy group included 45 patients, and the more‐than‐1‐year adjuvant therapy group included 77 patients. In the > 1‐year adjuvant therapy group, 7 patients discontinued therapy at 18 months and 7 patients at 2 years, none of whom experienced recurrence prior to discontinuation. The remaining patients in this group who had not developed recurrence were still receiving therapy at the end of the follow‐up period. There were no significant differences in any baseline characteristics between the 1‐year and more‐than‐1‐year adjuvant therapy groups.

### 3.2 Treatment Profiles

The median follow‐up time was 39.0 months (95% CI, 36.8–41.2) in the 1‐year adjuvant therapy group and 25.0 months (95% CI, 19.6–30.4) in the more‐than‐1‐year adjuvant therapy group. In the 1‐year adjuvant therapy group, 17 patients (37.8%) experienced recurrence after completing 1 year of therapy. In the more‐than‐1‐year adjuvant therapy group, 9 patients (11.7%) experienced recurrence; of these, 2 relapsed after completing 18 months of therapy and the remaining cases occurred during adjuvant therapy beyond 1 year. Among these patients, nine developed isolated locoregional recurrence (6 in the 1‐year adjuvant therapy group and 3 in the more‐than‐1‐year adjuvant therapy group), whereas 17 developed distant recurrence (11 in the 1‐year group and 6 in the more‐than‐1‐year group). The most common site of distant recurrence was the brain (1 in the 1‐year adjuvant therapy group and 4 in the more‐than‐1‐year adjuvant therapy group), followed by the lungs (3 in the 1‐year group and 1 in the more‐than‐1‐year group).

### 3.3 Survival Outcomes

**FIGURE 1:**
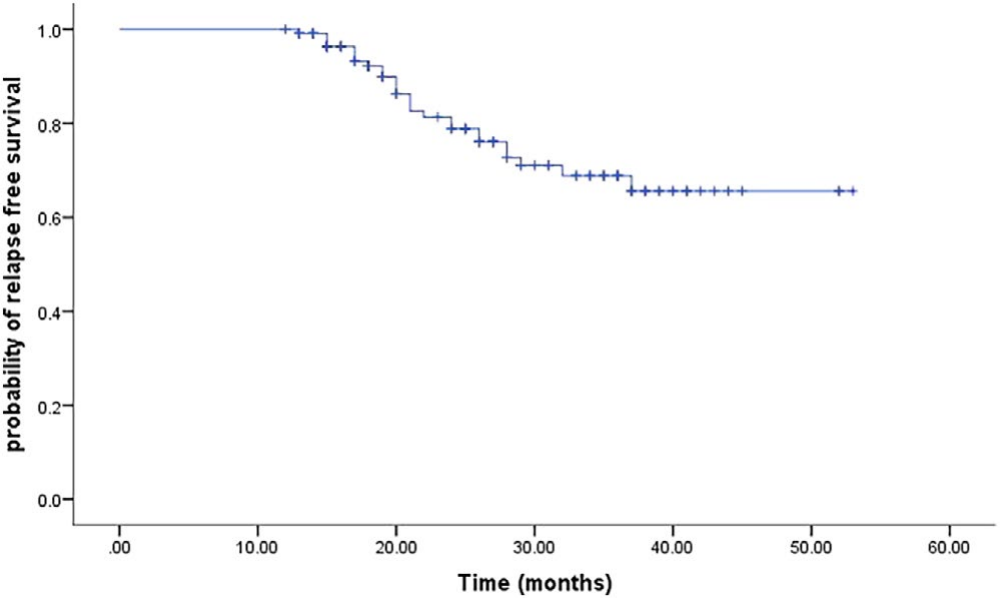
Relapse‐free survival of all included patients.

**FIGURE 2:**
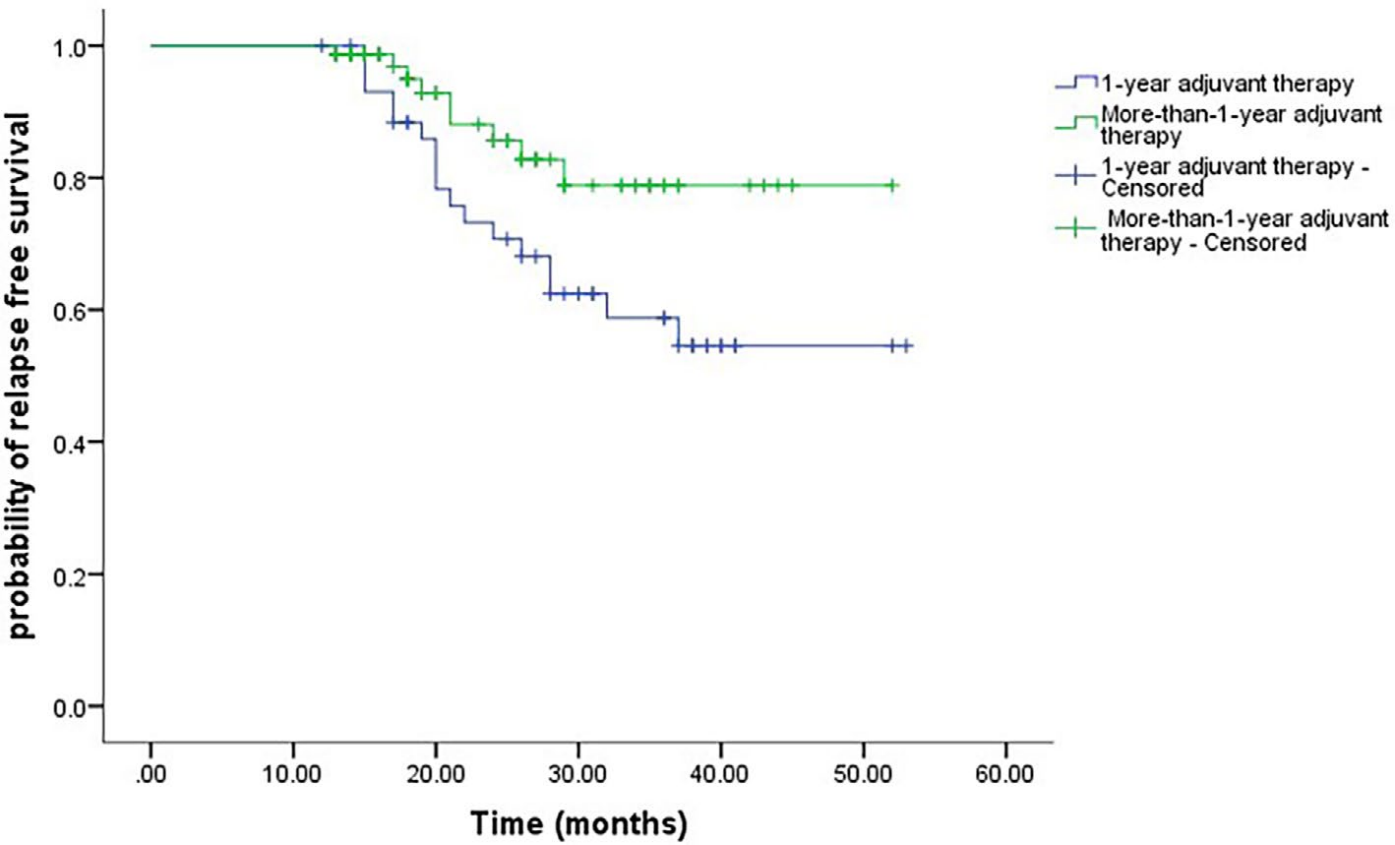
Relapse‐free survival stratified 1‐year adjuvant therapy and the more‐than‐1‐year adjuvant therapy (log‐rank *p* = 0.04).

As shown in Figure 1, among all patients, the 2‐year RFS rate was 78.9% (95% CI, 74.5–83.3), whereas the 3‐year RFS rate was 68.9% (95% CI, 63.5–74.3). A survival analysis stratified by treatment duration indicated that patients receiving more than 1 year of adjuvant therapy experienced significantly better RFS compared with those treated for only 1 year (Figure 2, log‐rank *p* = 0.04). In the multivariate analysis, an adjuvant therapy duration longer than 1 year was independently associated with improved recurrence‐free survival (HR, 2.42; 95% CI, 1.06–5.48; *p* = 0.035). Patients with more advanced (IIIC–D) disease showed a potential trend toward poorer recurrence‐free survival (HR, 1.42; 95% CI, 0.84–2.39; *p* = 0.188), although this difference did not reach statistical significance (Table 2). At the data cutoff, only three deaths had occurred in the 1‐year cohort and four deaths in the more‐than‐1‐year cohort, leaving an event count too small for a meaningful OS analysis.

**TABLE 2:** Univariate and multivariate analyses of relapse‐free survival.

	Univariate Cox analysis		Multivariate Cox analysis	
	HR (95% CI)	*p*	HR (95% CI)	*p*
Sex				
Male	Reference			
Female	0.70 (0.32–1.53)	0.377		
Age				
≥ 60 year	Reference			
< 60 year	0.79 (0.36–1.74)	0.557		
Disease stage				
III B	Reference		Reference	
III C–D	1.31 (0.81–2.12)	0.268	1.42 (0.84–2.39)	0.188
Subtype				
Cutaneous	Reference			
Acral	1.42 (0.63–3.22)	0.398		
Treatment duration				
More‐than 1 year	Reference		Reference	
1 year	2.27 (1.01–5.11)	0.048	2.42 (1.06–5.48)	0.035

Abbreviations: CI ‐ confidence intervals, HR ‐ hazard ratio.

### 3.4 Toxicity Profiles

Detailed toxicity profiles are presented in Table 3. No statistically significant differences in the incidence of toxicities were observed between the 1‐year adjuvant therapy group and the more‐than‐1‐year adjuvant therapy group. In the 1‐year group, four patients (8.89%) required dose adjustments due to adverse events, compared to seven (9.09%) in the more‐than‐1‐year group. Similarly, three patients (6.67%) in the 1‐year group and six (7.79%) in the more‐than‐1‐year group discontinued treatment because of adverse events. Notably, all toxicities that led to dose adjustments or treatment discontinuation in the more‐than‐1‐year group occurred within the first year of dabrafenib plus trametinib initiation.

**TABLE 3:** Safety profile of 1‐year adjuvant therapy group and the more‐than‐1‐year adjuvant therapy group.

Adverse event	1‐year adjuvant therapy group (*n* = 45)		More‐than‐1‐year adjuvant therapy group (*n* = 77)	
	Any grade	Grade 3 or 4	Any grade	Grade 3 or 4
Any adverse event	32.0 (71.11%)	13.0 (28.89%)	56 (72.73%)	25 (32.47%)
Pyrexia	27.0 (60.00%)	10.0 (22.22%)	46 (59.74%)	20 (25.97%)
Fatigue	15.0 (33.33%)	5.0 (11.11%)	23 (29.87%)	6 (7.79%)
Nausea	13.0 (28.89%)	3.0 (6.67%)	21 (27.27%)	5 (6.49%)
Headache	11.0 (24.44%)	3.0 (6.67%)	19 (24.68%)	4 (5.19%)
Chills	8.0 (17.78%)	2.0 (4.44%)	19 (24.68%)	4 (5.19%)
Diarrhea	7.0 (15.56%)	2.0 (4.44%)	14 (18.18%)	3 (3.90%)
Rash	5.0 (11.11%)	1.0 (2.22%)	13 (16.88%)	2 (2.60%)
Vomiting	5.0 (11.11%)	0	10 (12.99%)	0
Arthralgia	4.0 (8.89%)	0	7 (9.09%)	1 (1.30%)
Panniculitis	4.0 (8.89%)	0	5 (6.49%)	0

## 4 Discussion

The optimal duration of adjuvant therapy for melanoma remains uncertain, with no evidence supporting additional benefit from prolonged treatment. In COMBI‐AD, as in BRIM8, KEYNOTE‐054, CheckMate 238, and CheckMate 915, adjuvant dabrafenib plus trametinib was administered for 12 months. The common 1‐year duration of adjuvant melanoma therapy likely reflects the precedent set by the Kirkwood trial, which established a 1‐year standard for adjuvant interferon treatment, though no trials explicitly justify this duration [10].

The potential of immunotherapy for prolonged responses posttreatment suggests shorter adjuvant immunotherapy durations may be feasible. Experts advocate for noninferiority trials comparing 6‐ and 12‐month regimens to address this evidence gap [11]. Unlike immunotherapy, the COMBI‐AD trial revealed frequent melanoma recurrences within the first year after discontinuing adjuvant TT [5]. Many physicians in China favor extending the duration of adjuvant TT to prevent early recurrence.

Introduced in 2000, imatinib (IM) markedly improved prognosis for gastrointestinal stromal tumor (GIST) patients. The ACOSOG Z9001 trial showed that 1‐year adjuvant IM enhances RFS but not OS in high‐risk, KIT‐positive GIST patients post resection, leading to national guidelines endorsing a 1‐year regimen [12]. Recurrence of GISTs frequently occurs in the initial years after ceasing adjuvant IM, indicating that a 12‐month treatment duration may be insufficient [12]. Subsequently, the Phase III SSG XVIII/AIO trial by Joensuu et al., with 400 patients, evaluated extending IM therapy to 3 years, revealing significantly better RFS and OS (HR for RFS = 0.46, *p* < 0.001; HR for OS = 0.45, *p* = 0.019) [13]. These results prompted FDA approval of a 3‐year adjuvant IM regimen in January 2012 [13]. In recent years, Korean investigators have conducted real‐world studies evaluating a 5‐year adjuvant IM regimen, with preliminary results indicating that the 5‐year treatment group demonstrates superior RFS compared to the 3‐year group [14].

In this registry‐based retrospective study, we evaluated the safety and efficacy of more‐than‐1‐year adjuvant dabrafenib plus trametinib, compared with a 1‐year regimen, among patients with resected Stage III melanoma. The extended adjuvant therapy demonstrated superior recurrence‐free survival (log‐rank *p* = 0.04) and was associated with a reduced risk of recurrence in multivariate analysis (HR, 2.42; *p* = 0.035). However, the statistical significance was only marginal, potentially attributable to the shorter follow‐up period and the limited number of events in the analysis. Furthermore, adverse event rates did not significantly differ between the extended regimen and the 1‐year regimen. To our knowledge, this is the first study to highlight the feasibility of prolonging adjuvant dabrafenib plus trametinib in resected Stage III melanoma, which may provide a basis for future prospective research.

Although baseline characteristics were broadly similar, patients remaining on therapy > 1 year were marginally younger. During the latter half of accrual, five of the six participating centers revised their protocols to “treat until progression,” while one center continued the original 12‐month stop rule; this institutional divergence, together with center‐specific imaging intervals and follow‐up intensity, could have influenced the timing of detected recurrences. Median follow‐up also differed (39 months in the 12‐month cohort vs. 25 months in the extended‐therapy cohort), potentially biasing event capture.

Since 2019, dabrafenib plus trametinib has been implemented as adjuvant therapy for Chinese patients with resected Stage III melanoma. In early cohorts, recurrence rates increased after completing 1 year of adjuvant treatment. Consequently, some centers attempted extending the duration of adjuvant therapy to 1.5 or 2 years, while others plan to continue therapy for up to 3 years. The longest treatment duration observed thus far has reached 35 months without discontinuation. Therefore, the more‐than‐1‐year adjuvant therapy group includes patients who stopped therapy after 1.5 years, those who stopped after 2 years, and those still undergoing therapy who have not yet reached 3 years of treatment.

The potential for resistance to systemic therapy in advanced stages is an important consideration when selecting adjuvant regimens. Patients relapsing after adjuvant TT typically achieve favorable outcomes with subsequent anti‐PD‐1 immunotherapy, comparable to those in first‐line or therapy‐naïve Stage IV melanoma [15]. Prolonged adjuvant TT does not appear to compromise the efficacy of subsequent immune checkpoint inhibitors (ICIs). However, the response rate to TT rechallenge is approximately 25% [15]. It remains unclear whether extending the duration of adjuvant TT might increase resistance and thereby diminish the potential benefits of TT rechallenge in relapsed patients.

Common adverse events and their frequencies did not differ significantly between the 1‐year adjuvant therapy group and the more‐than‐1‐year adjuvant therapy group. Furthermore, all patients who discontinued treatment or underwent dose adjustments due to toxicity did so within the first year of initiating dabrafenib plus trametinib. These observations suggest that extending dabrafenib plus trametinib therapy does not increase the incidence of adverse events.

Several limitations of this analysis should be noted. Although the study was multicenter, its retrospective design may expose the findings to selection bias. In particular, the patient classification was not determined prospectively, which may limit both the interpretation and the generalizability of our results. In addition, the extended‐therapy cohort was slightly younger, and intercenter variations in treatment duration, follow‐up strategy, and observation time may still introduce residual confounding. Another limitation is the relatively small sample size. Nonetheless, given the rarity of melanoma in East Asian populations, the number of patients included here exceeded that in our previous investigations as well as in comparable Japanese studies [9, 16]. Finally, while both longer follow‐up and prospective clinical trials are necessary to determine whether the observed recurrence‐free survival benefit from more‐than‐1‐year adjuvant therapy ultimately translates into an OS advantage, it remains equally important to identify which patient populations are most likely to benefit from prolonged treatment regimens.

In conclusion, extending adjuvant dabrafenib plus trametinib beyond 1 year in Chinese patients with resected Stage III melanoma may be feasible and is associated with favorable RFS outcomes and manageable toxicity profiles. These findings warrant further validation in future investigations.

## Ethics Statement

All procedures performed in studies involving human participants were in accordance with the ethical standards of the institutional and/or national research committee and with the 1964 Helsinki Declaration and its later amendments or comparable ethical standards.

## Consent

Informed consent was obtained from all individual participants included in the study.

## Conflicts of Interest

The authors declare no conflicts of interest.

## Data Availability

The data that support the findings of this study are available from the corresponding author upon reasonable request.
